# Identification and characterization of a novel 6′-N-aminoglycoside acetyltransferase AAC(6′)-Va from a clinical isolate of *Aeromonas hydrophila*

**DOI:** 10.3389/fmicb.2023.1229593

**Published:** 2023-10-18

**Authors:** Guozhi Zhang, Lei Zhang, Yuning Sha, Qiaoying Chen, Naru Lin, Jingxuan Zhao, Yuan Zhang, Yongan Ji, Weiyan Jiang, Xueya Zhang, Qiaoling Li, Junwan Lu, Xi Lin, Kewei Li, Hailin Zhang, Qiyu Bao, Jun Lu, Yunliang Hu, Tingting Zhu

**Affiliations:** ^1^The Second Affiliated Hospital and Yuying Children’s Hospital, Wenzhou Medical University, Wenzhou, China; ^2^Key Laboratory of Medical Genetics of Zhejiang Province, Key Laboratory of Laboratory Medicine, Ministry of Education, School of Laboratory Medicine and Life Sciences, Wenzhou Medical University, Wenzhou, China; ^3^Department of Clinical Laboratory, Quzhou People’s Hospital, The Quzhou Affiliated Hospital of Wenzhou Medical University, Quzhou, China; ^4^Medical Molecular Biology Laboratory, School of Medicine, Jinhua Polytechnic, Jinhua, China

**Keywords:** AAC(6′)-Va, aminoglycoside resistance, aminoglycoside-modifying enzyme, aminoglycoside 6′-acetyltransferase, *Aeromonas hydrophila*

## Abstract

**Background:**

*Aeromonas* species have been identified as agents responsible for various diseases in both humans and animals. Multidrug-resistant *Aeromonas* strains pose a significant public health threat due to their emergence and spread in clinical settings and the environment. The aim of this study was to determine a novel resistance mechanism against aminoglycoside antimicrobials in a clinical isolate.

**Methods:**

The function of *aac(6′)-Va* was verified by gene cloning and antibiotic susceptibility tests. To explore the *in vivo* activity of the enzyme, recombinant proteins were expressed, and enzyme kinetics were tested. To determine the molecular background and mechanism of *aac(6′)-Va*, whole-genome sequencing and bioinformatic analysis were performed.

**Results:**

The novel aminoglycoside *N*-acetyltransferase gene *aac(6′)-Va* confers resistance to several aminoglycosides. Among the antimicrobials tested, ribostamycin showed the highest increase (128-fold) in the minimum inhibitory concentration (MIC) compared with the control strains. According to the MIC results of the cloned *aac(6′)-Va*, AAC(6′)-Va also showed the highest catalytic efficiency for ribostamycin [*k*_cat_/*K*_m_ ratio = (3.35 ± 0.17) × 10^4^ M^−1^ s^−1^]. Sharing the highest amino acid identity of 54.68% with AAC(6′)-VaIc, the novel aminoglycoside *N*-acetyltransferase constituted a new branch of the AAC(6′) family due to its different resistance profiles. The gene context of *aac(6′)-Va* and its close relatives was conserved in the genomes of species of the genus *Aeromonas*.

**Conclusion:**

The novel resistance gene *aac(6′)-Va* confers resistance to several aminoglycosides, especially ribostamycin. Our finding of a novel resistance gene in clinical *A. hydrophila* will help us develop more effective treatments for this pathogen’s infections.

## Introduction

*Aeromonas* spp., facultative anaerobic and rod-shaped bacterial species, are widely present in environments, especially in aquatic media, and are increasingly important for causing various clinical infections, including diarrhea, soft tissue infections, bacteremia, and gastroenteritis (Chauret et al., 2001; Syue et al., 2016). Many infections caused by *Aeromonas* spp. are self-limiting. However, in patients who have severe underlying diseases or in immunocompromised individuals, invasive infections can be urgent and develop rapidly. The reported mortality rates among patients with *Aeromonas* bacteremia range from 24 to 63% (Chen et al., 2014; Pessoa et al., 2019). Due to the similar clinical manifestations of *Vibrio* and *Aeromonas* infections, they are often misdiagnosed as *Vibrio* infections prior to microbiological identification in the laboratory, which may lead to improper use of antimicrobials and ineffective treatment (Syue et al., 2016).

Pathogenic bacteria play a significant role in the occurrence of common fish diseases in aquaculture (Rosa et al., 2019). Among the identified bacterial pathogens, *A. hydrophila* is widely recognized as a major pathogen affecting various aquatic animal species and has been responsible for significant economic losses in recent years (Saraceni et al., 2016; Pengcheng et al., 2017). The use of antimicrobials is the main factor for the emergence of resistance in *A. hydrophila*. Multidrug-resistant *A. hydrophila* strains from other regions of the world have been isolated. It has been reported that all clinical isolates of *A. hydrophila* exhibited innate resistance to ampicillin, amoxicillin, amoxicillin–clavulanic acid, ampicillin–sulbactam, and cefoxitin. Additionally, *A. hydrophila* was intrinsically resistant to benzylpenicillin, glycopeptides, lipoglycopeptides, fusidic acid, lincosamides, streptogramins, rifampicin, oxazolidines, and macrolides (except azithromycin) (Chacón et al., 2023).

Multidrug resistance in pathogenic bacteria is typically mediated by acquired resistance. Resistance determinants are often related to mobile genetic elements (MGEs), such as integrons, transposons and plasmids, which facilitate their rapid spread (Romero et al., 2012). The transfer of these mobile genetic elements occurs via DNA transfer mechanisms, including transformation, transduction, and conjugation, in bacteria. *Aeromonas hydrophila*, a common pathogenic bacterium, exhibits resistance to multiple antibiotics, which can be chromosomally mediated or attributed to the acquisition of plasmids or integrons (Stratev and Odeyemi, 2016). Environmental antimicrobials can promote horizontal gene transfer (HGT) between bacteria, causing an escalation of bacterial antibiotic resistance and posing significant public health risks (Skwor et al., 2020).

Aminoglycoside antibiotics are important anti-infective agents due to their broad-spectrum antimicrobial activity and ability to work synergistically with other antibiotics. These antibiotics interfere with bacterial protein synthesis, inducing mistranslation of proteins and altering the integrity of bacterial membranes. Aminoglycoside-modifying enzymes can be grouped into three types according to their modification sites: aminoglycoside O-phosphotransferases (APHs), aminoglycoside O-nucleotidyltransferases (ANTs), and aminoglycoside N-acetyltransferases (AACs). AACs use acetyl-coenzyme A (acetyl-CoA) as a substrate and transfer the acetyl group to an amine group of aminoglycosides for modification. Based on the site of regioselective modification of aminoglycosides, AACs are divided into four subclasses: AAC(1), AAC(2′), AAC(3), and AAC(6′).

This study reports on the identification and characterization of a newly discovered aminoglycoside 6′-N-acetyltransferase, AAC(6′)-Va, which is encoded in the chromosome of an *A. hydrophila* isolate obtained from a clinical sample. Furthermore, we used sequence analysis to investigate the genetic context of the *aac(6′)-Va* gene and its relationship with other aac genes.

## Materials and methods

### Bacterial strains and plasmids

For this study, we collected samples from patients with different infectious diseases to investigate the antimicrobial resistance of multidrug-resistant (MDR) *Aeromonas* in a clinical setting. *A. hydrophila* QZ124 was obtained from the urine of a male patient with traumatic urethral rupture at the Urology Department of Quzhou Affiliated Hospital of Wenzhou Medical University in southeastern China in 2021. After identification using the Vitek-60 microorganism autoanalysis system (BioMerieux corporate, Craponne, France), the isolate was confirmed as *A. hydrophila* through analysis of the 16S rRNA gene sequence and whole-genome average nucleotide identity (ANI) analysis using FastANI (Jain et al., 2018). Information on the strains and plasmids used in this investigation is provided in Table 1.

**Table 1:** Bacteria and plasmids used in this work.

Strain or plasmid	Relevant characteristic(s)	Reference or source
Strain		
QZ124	The wild-type strain of *Aeromonas hydrophila* QZ124	This study
DH5α	*E. coli* DH5α was used as a host for the cloning of the *aac(6′)-Va* gene	Our laboratory collection
BL21 (DE3)	*E. coli* BL21 was used as a host for expression of AAC(6′)-Va	Our laboratory collection
ATCC 25922	*E. coli* ATCC 25922 was used as a quality control for antimicrobial susceptibility testing	Our laboratory collection
pMD19-*aac(6′)*-*Va*/DH5α	DH5α carrying the recombinant plasmid pMD19-*aac(6′)-Va*	This study
pCold I-*aac(6′)*-*Va*/BL21	BL21 carrying the recombinant plasmid pCold I-*aac(6′)-Va*	This study
Plasmid		
pMD19/DH5α	Cloning vector for the PCR products of the *aac(6′)-Va* gene with its upstream promoter region, ampicillin resistance	Our laboratory collection
pCold I/BL21	Expression vector for the PCR products of the ORF of the *aac(6′)-Va* gene, ampicillin resistance	Our laboratory collection

### Whole-genome sequencing and functional analysis

An AxyPrep Bacterial Genomic DNA Miniprep Kit (Axygen Scientific, Union City, CA, United States) was used for the extraction of DNA from *A. hydrophila* QZ124. DNA sequencing was carried out by Shanghai Personal Biotechnology Co., Ltd. (Shanghai, China) using both the Illumina HiSeq-2500 and PacBio RS II platforms. PacBio long reads were initially assembled with SPAdes v3.14.1 (Bankevich et al., 2012), followed by mapping of short reads to the draft whole-genome assembly with Pilon v1.23 to improve the quality of the draft genome assembly (Walker et al., 2014). ORFs present in the genome sequence were predicted by Prokka v1.14.6 (Seemann, 2014), while BLAST analysis against the protein sequence database of the NCBI helped annotate their function with an e-value threshold of 1e-5. Antimicrobial resistance genes were identified utilizing the Resistance Gene Identifier v5.2.0 (available at https://github.com/arpcard/rgi) along with the comprehensive antibiotic resistance database (CARD, McArthur et al., 2013). ANI was calculated with FastANI v1.31 (Jain et al., 2018). The genomic features were visualized by GView Server (Petkau et al., 2010). The comparative genomic analysis was performed by means of clinker v0.0.24 (Gilchrist and Chooi, 2021). The promoter region of *aac(6′)-Va* was predicted by BPROM.^1^ AAC(6′)-Va molecules were analyzed using the ExPASy ProtParam Tool to determine their molecular weight and pI values.^2^ MAFFT v7.475 (Katoh and Standley, 2013), MEGAX (Kumar et al., 2018) and ggtree v3.2.0 were used to align the amino acid sequences and construct neighbor-joining phylogenies for AAC(6′)-Va and other AACs. A CD search^3^ was used to discover the conserved domain of AAC(6′)-Va. The sequence retrieval and other bioinformatic tools were written in Python.^4^

### Cloning of the *aac(6′)-Va* gene

For amplification of the upstream promoter region and *aac(6′)-Va*, we utilized PCR with primers flanked by *Bam*HI and *Hind*III restriction endonuclease adaptors at the 5′ and 3′ ends (Takara Bio, Inc., Dalian, China). *Bam*HI and *Hind*III enzymes were used to digest the resulting PCR product, which was then ligated into the pMD19 vector using a T4 DNA ligase cloning kit from Takara Bio, Inc. (Dalian, China). After rendering *E. coli* DH5α cells competent through the calcium chloride method (Chan et al., 2013), the cells were transformed with the recombinant plasmid pMD19-pro-*aac(6′)-Va* and selected on Luria-Bertani agar plates supplemented with 100 μg/mL ampicillin. To confirm the cloned insert sequence of *aac(6′)-Va* and its upstream promoter region in the recombinant plasmid, Sanger sequencing and restriction enzyme digestion were employed (Shanghai Sunny Biotechnology Co., Ltd., Shanghai, China) (Table 2).

**Table 2:** Primers for cloning the *aac(6′)-Va* gene.

Primer^a^	Sequence (5′ → 3′)	Restriction endonuclease	Vector	Annealing temperature (°C)	Amplicon size (bp)
pro-*aac(6′)-Va*-F	GAGCGGCTGGTGGTGGATTTCAGCC		pMD19	60	785
pro-*aac(6′)-Va* -R	CGAGCCTGTGAGAAACAAACAAGGCCCTGG		pMD19	60	785
orf-*aac(6′)-Va* –F	CGGATCCGGACGACGACGACAAGATGACGAACGCCGACTGGCGGATAG	*Bam*HI + Enterokinase	pColdI	58	491
orf-*aac(6′)-Va* –R	CAAGCTTGCGAGCCTGTGAGAAACAAACAAGGCCCTGG	*Hind*III	pColdI	58	491

a Primers starting with “pro” were used to clone the aac(6′)-Va gene and its promoter region; primers starting with “orf” were used to clone the ORF of the aac(6′)-Va gene.

### Antimicrobial susceptibility testing

Suspensions of *A. hydrophila* QZ124, pMD19/DH5α, pMD19-*aac(6′)-Va*/DH5α and DH5α with a McFarland standard value of 0.5 were prepared and inoculated onto Mueller–Hinton agar plates to determine antibiotic susceptibility. All tested antimicrobials in this work were listed in Table 3, including ten aminoglycosides (gentamicin, tobramycin, paromomycin, neomycin, streptomycin, sisomicin, ribostamycin, amikacin, spectinomycin and kanamycin); nine β-lactams (penicillinG, ampicillin, cefoxitin, cefazolin, cefatriaxone, cefotaxime, ceftazidime, aztreonam and meropenem) and one polymyxin (polymyxin B). The plates were incubated at 37°C for 16 h, and the minimum inhibitory concentration (MIC) was interpreted according to the Clinical and Laboratory Standards Institute (CLSI) breakpoint criteria for *Enterobacteriaceae*. *Escherichia coli* ATCC 25922 was used as the MIC reference strain for quality control. The test was repeated three times to ensure accuracy.

**Table 3:** MIC values of various antimicrobials for five bacterial strains (μg/mL).

Antimicrobial class	Antimicrobial	*A.hydrophila* QZ124	pMD19-*aac(6′)-Va*/DH5α	pMD19/DH5α	DH5α	ATCC25922
Aminoglycosides	Gentamicin^a^^b^	64	1	0.5	8	0.5
	Tobramycin^a^^b^	64	8	0.5	0.5	0.5
	Paromomycin	2	4	4	8	4
	Neomycin	1	1	8	16	1
	Streptomycin^a^	128	4	4	4	4
	Sisomicin^a^^b^	32	64	2	2	2
	Ribostamycin^a^^b^	2048	256	2	2	4
	Amikacin	8	1	1	1	2
	Spectinomycin^a^	128	8	8	32	8
	Kanamycin^a^^b^	256	64	2	2	4
β-Lactams	PenicillinG^a^	>2048	/	/	/	32
	Ampicillin^a^	>1,024	/	/	/	8
	Cefoxitin	16	/	/	/	4
	Cefazolin^a^	>256	/	/	/	4
	Cefatriaxone	16	/	/	/	0.125
	Cefotaxime	32	/	/	/	0.125
	Ceftazidime^a^	128	/	/	/	0.5
	Aztreonam^a^	32	/	/	/	0.125
	Meropenem	0.125	/	/	/	0.03
Polymyxins	Polymyxin B^a^	512	/	/	/	1

“/” indicates that the susceptibility test was not performed.

a A.hydrophila QZ124 is intrinsically resistant to these antimicrobial agents.

b pMD19-aac(6′)-Va/DH5α is intrinsically resistant to these antimicrobial agents.

### Expression and purification of recombinant AAC(6′)-Va

Using the orf-*aac(6′)-Va* primers listed in Table 2, the ORF of the *aac(6′)-Va* gene was PCR-amplified and cloned and inserted into the pCold I vector between the *Bam*HI and *Hind*III restriction sites (Qing et al., 2004). The resulting recombinant plasmid, pCold I-*aac(6′)-Va*, was transformed into *E. coli* BL21 competent cells and screened on LB agar plates containing 100 μg/mL ampicillin (pCold I-*aac(6′)-Va*/BL21). The presence of the *aac(6′)-Va* gene in the transformant was verified by PCR and PCR product sequencing. The overnight culture of the recombinant strain was grown in LB medium containing 100 μg/mL ampicillin, and IPTG was added to a final concentration of 0.1 mM when the OD600 of the culture reached 0.6. The induced culture was further incubated at 16°C for 20 h. The cells were sonicated, and the recombinant protein was purified using a His-tag Protein Purification Kit (Beyotime, Shanghai, China). The His-tag was removed from the sample using enterokinase, and the presence of *aac(6′)-Va* was confirmed by SDS-PAGE and Coomassie Brilliant Blue staining. The protein concentration was analyzed by a BCA protein assay kit from Thermo Fisher Scientific (Rockford, IL, United States). To determine the approximate range of molecular weights, ultrafiltration was performed using centrifugal filter units with pore sizes of 10 kDa, 30 kDa, 50 kDa, and 100 kDa (Millipore, Amicon Ultra0.5). The quaternary structure of AAC(6′)-Va was examined by clear-native PAGE. Bovine serum albumin (BSA, 66.4 kDa, pI: 4.7) was used as the protein marker for clear-native PAGE (Wittig and Schägger, 2005). Without protein denaturants, 10% clear-native PAGE was used to separate AAC(6′)-Va and the marker. The samples were electrophoresed at 120 V for 30 min, followed by 160 V for 45 min to separate the target protein and the corresponding marker.

### Kinetic studies of AAC(6′)-Va

To study the activity of AAC(6′)-Va, we measured its kinetic parameters spectrophotometrically based on the production of coenzyme A (CoASH) resulting from the transfer of the acetyl moiety to the aminoglycoside. We used a wavelength of 412 nm to determine the increase in absorbance resulting from the reaction between CoASH’s thiol group and DTNB, which forms pyridine-4-thiolate (TNB). TNB was subsequently replaced with dithiodipyridine. This method was previously reported (Hegde et al., 2001; Galimand et al., 2015). The kinetic assays were performed in a 200 μL reaction mixture including acetyl-CoA (80 μM), 5,5′-dithiobis(2-nitrobenzoic acid) (DTNB) (2 mM), 2-(N-morpholino) ethanesulfonic acid (MES) (25 mM, pH 6.0), ethylenediamine tetraacetic acid (EDTA) (1 mM), and varying concentrations of aminoglycosides (5–800 μM) (Franklin and Clarke, 2001). We initiated the reactions by adding purified enzyme to a final concentration of 8.0 μg/mL and monitored them for 10 min at room temperature using a Synergy Neo2 Multi-Mode Microplate Reader (Biotek, VT, United States). We determined the steady-state kinetic parameters (*k*_cat_ and *K*_m_) using GraphPad Prism 9 (GraphPad Software, San Jose, CA, United States) through nonlinear regression analysis of the initial reaction rates with the Michaelis–Menten equation.

### Nucleotide sequence accession numbers

The GenBank accession numbers for the *aac(6′)-Va* gene, chromosome and pQZ124-211 of *A. hydrophila* QZ124 were OQ685298, CP121100, and CP121101, respectively.

## Results

### Classification and genome characteristics of *Aeromonas hydrophila* QZ124

**Figure 1:**
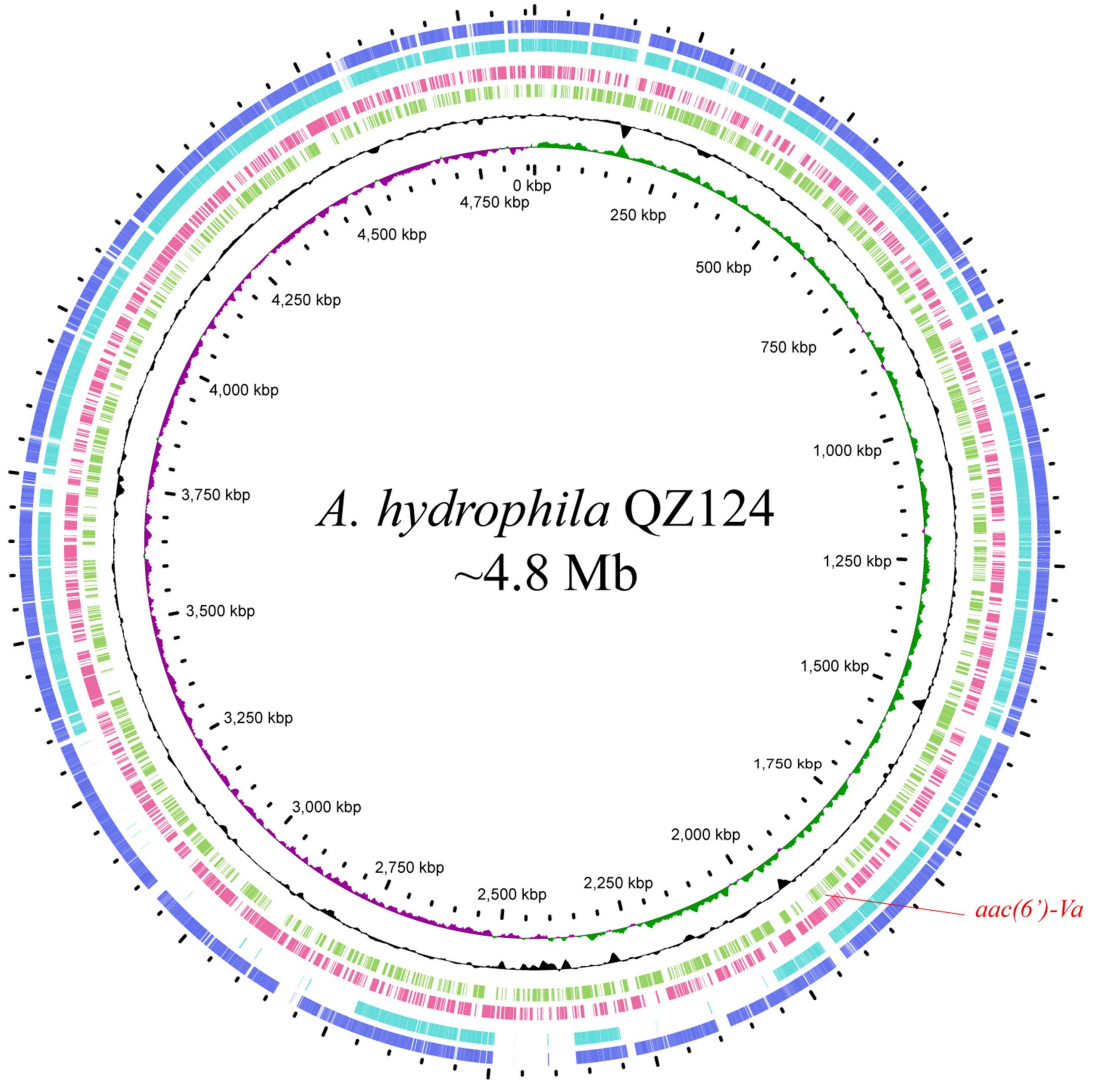
Genome map and comparison of the chromosome sequence of *A. hydrophila* QZ124 with other similar genomes with high identities. From outside to inside: circles 1 and 2 are homologous regions of the chromosomes of *A. hydrophila* ATCC7966 (NC_008570.1) and *A. hydrophila* WCX23 (CP028418.1) with *A. hydrophila* QZ124, while the unmatched regions are left blank; circles 3 and 4 display predicted ORFs encoded in the reverse and forward strands, and circles 5, 6, and 7 represent the GC content, GC skew, and scale in kb of the *A. hydrophila* QZ124 chromosome, respectively.

According to the homology analysis of 16S ribosomal RNA genes, QZ124 revealed the highest similarity to *A. hydrophila* WCX23 (CP028418.1), with an identity of 99.87 and 100% coverage. Furthermore, ANI analysis revealed that the genome sequence of QZ124 shared identity (96.77%) with that of the type strain *A. hydrophila* ATCC7966 (NC_008570.1), and this isolate was finally classified as *A. hydrophila* and thus designated *A. hydrophila* QZ124 (See Figure 1). The complete genome of *A. hydrophila* QZ124 contained a chromosome and a circular plasmid. The length of the chromosome was approximately 4.86 Mb, which encoded 5,079 open reading frames (ORFs). The plasmid, designated pQZ124-211, was 211,418 bp in length and encoded 268 ORFs (Table 4).

**Table 4:** General features of the *A. hydrophila* QZ124 genome.

	Chromosome	pQZ124-211
Size (bp)	4,863,637	211,418
GC content (%)	61.08	53.25
ORFs	5,079	268
Known protein	2,835	50
Hypothetical proteins	2,244	218
Protein coding (%)	97.13	100
Average ORF length (bp)	796.5	642
Average protein length (aa)	268.7	213
tRNAs	119	0
rRNA operons	(16S-23S-5S)*10	0

### Functional characteristics of the aac(6′)-Va gene

QZ124 showed resistance to 17 out of 20 antimicrobials tested, including aminoglycosides (such as gentamicin, spectinomycin, tobramycin, streptomycin, kanamycin, and sisomicin), β-lactams (ampicillin, ceftazidime, cefoxitin, meropenem and so on), and polymyxin B. According to the annotation results of the complete genome sequence, a total of 13 genes (from 13 genotypes) with ≥95.0% similarity to the antibiotic resistance genes in the comprehensive antibiotic resistance database (CARD, McArthur et al., 2013) were identified. They included three genotypes of aminoglycoside-modifying enzymes [*aph(3′)-Ia*, *aph(3″)-Ib*, and *aph(6)-Id*], seven genotypes of β-lactamase (*bla*_MOX-3_, *bla*_OXA-10_, *bla*_OXA-726_, *bla*_OXA-1_, *bla*_PER-3_, *imiH*, and *cepS*), quinolone resistance gene (*qnrVC4*), MFS efflux pump (*cmlA5*), and macrolide phosphotransferase (*mphA*). Notably, although the strain showed higher MIC levels to gentamicin (64 μg/mL) and tobramycin (64 μg/mL) (Table 3), no functionally characterized gene that conferred resistance to gentamicin and/or tobramycin was identified.

**Figure 2:**
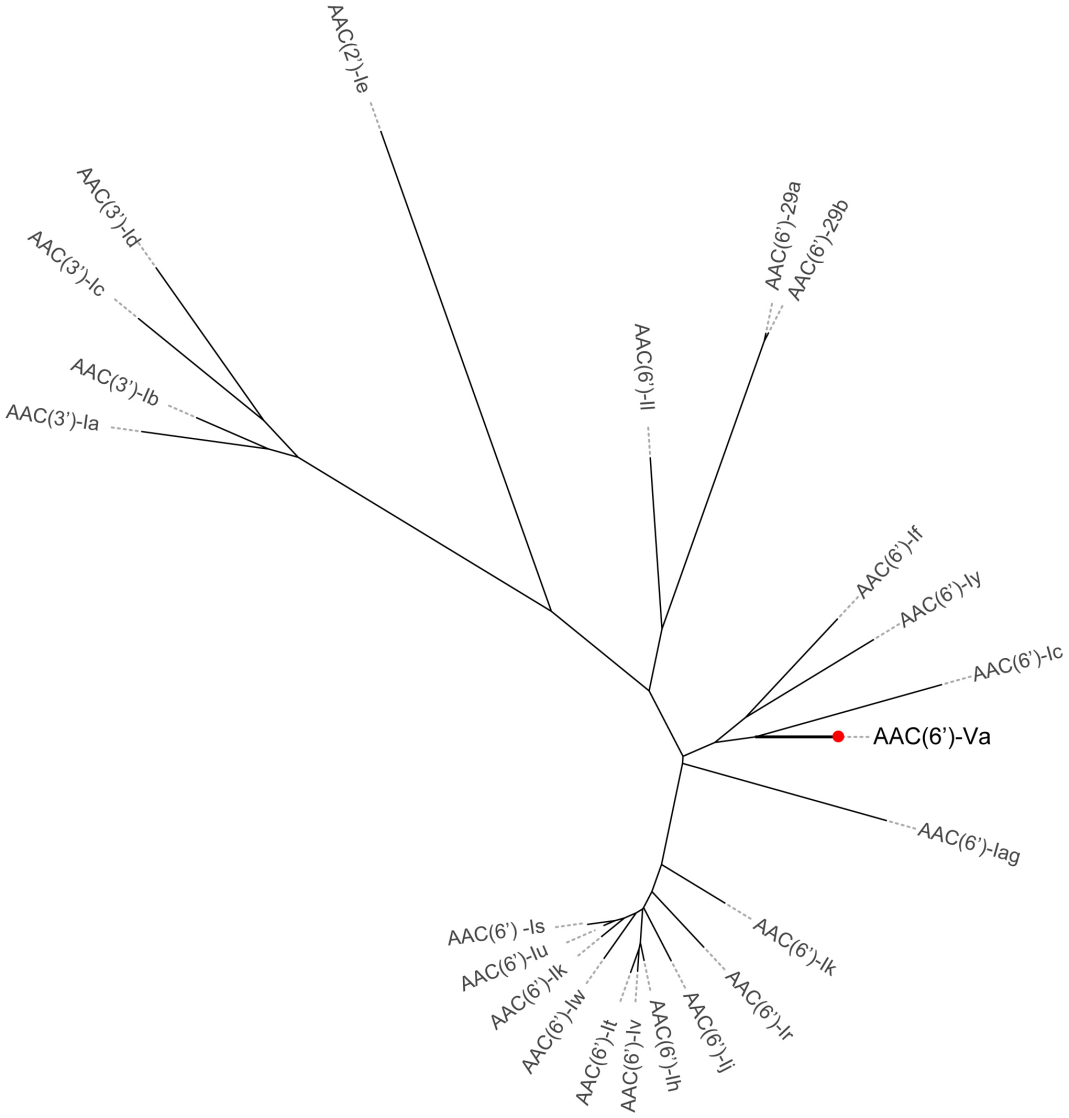
A phylogenetic tree showing the relationship of AAC(6′)-Va with other functionally characterized AACs. AAC(6′)-Va is highlighted with a red dot.

To investigate whether any novel aminoglycoside resistance gene conferring resistance to gentamicin and/or tobramycin was encoded in the *A. hydrophila* QZ124 genome, the annotation result of the genome sequence was checked, and one predicted *aac(6′)-Ic*-like gene was found (this gene was finally designated *aac(6′)-Va*) that shared the highest amino acid identity (54.68%) with a functionally characterized AAC(6′)-Ic (AAA26549.1) (Figure 2). To investigate the function of the *aac(6′)-Va* gene, the open reading frame (ORF) sequence encoding *aac(6′)-Va* and its promoter region were cloned and inserted into the pMD19 vector. Subsequently, the recombinant plasmid pMD19-*aac(6′)-Va* was transformed into *E. coli* DH5α for further analysis. The MIC of the transformant harboring pMD19-*aac(6′)-Va*/DH5α against several aminoglycoside antibiotics was determined, and the MIC levels to ribostamycin, sisomicin, kanamycin, tobramycin, and gentamicin increased 128-, 32-, 32-, 16-, and 2-fold, respectively, in comparison with those for the control strains (DH5α or DH5α carrying the vector pMD19) (Table 3). However, no change in the MIC level to streptomycin or amikacin was observed.

### Comparative and functional analysis of the novel aminoglycoside resistance 6′-N-acetyltransferase AAC(6′)-Va with its homologs

**Figure 3:**
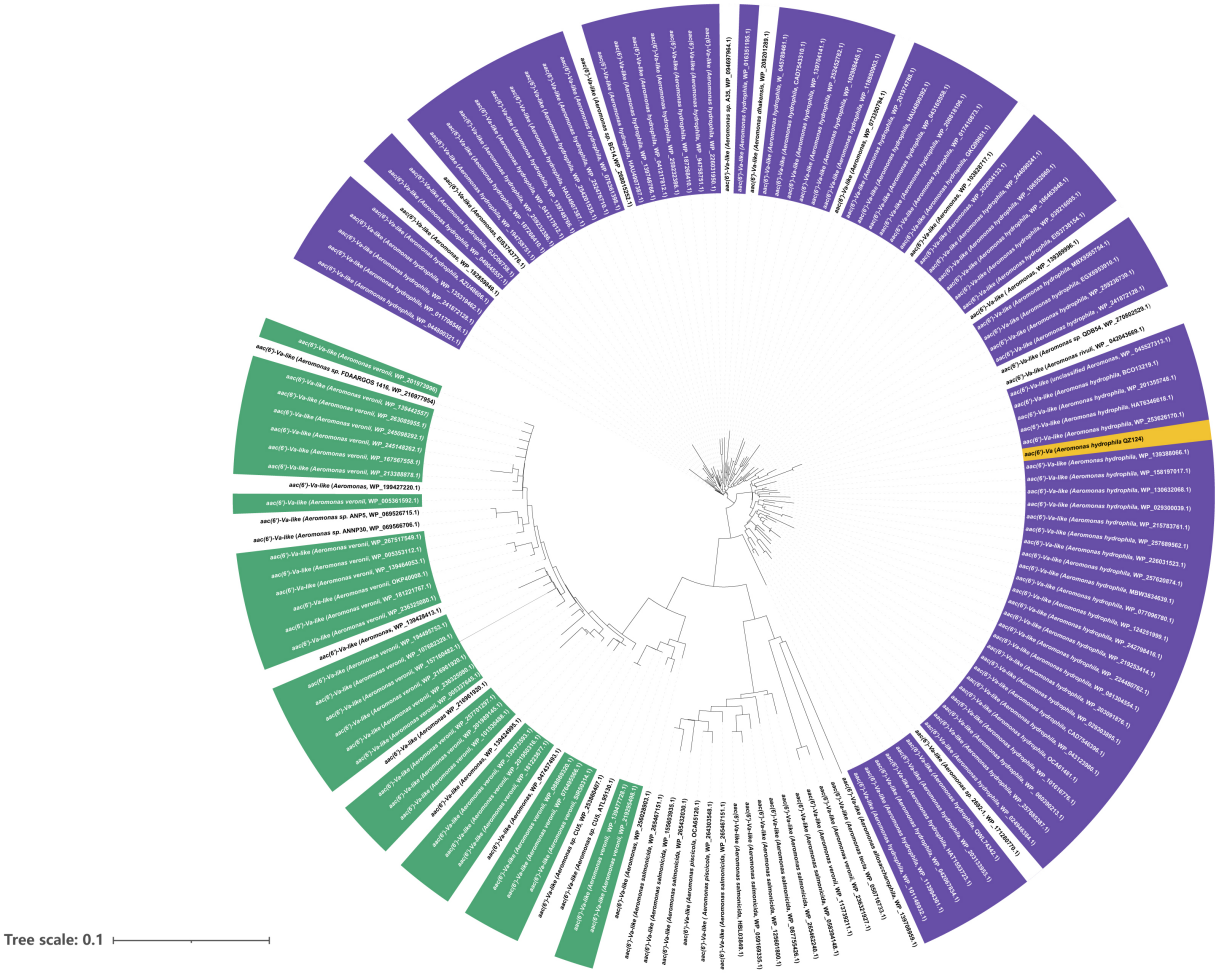
A phylogenetic tree showing the relationship of *aac(6′)-Va* with other putative *aac(6′)-Va*-like genes. *Aac(6′)-Va* is highlighted with a yellow box. The purple part represents *Aeromonas hydrophila*. Green represents *Aeromonas veronii*, and white represents other *Aeromonas* species.

AAC(6′)-Va was a 459 bp long gene encoding a 152 amino acid protein of 16.7 kDa with a pI value of 6.06. Furthermore, the enzyme was overexpressed (Supplementary Figure S1) and purified (Supplementary Figure S2). The results of ultrafiltration showed that AAC(6′)-Va remained in the upper layer of the 100 kDa filtrate, indicating that its molecular weight was greater than 100 kDa (Supplementary Figure S3). The results of clear-native PAGE revealed three bands with large molecular weights, suggesting that AAC(6′)-Va may not exist in a single polymer form but in three different polymer forms (Supplementary Figure S4). A total of 150 *aac(6′)-Va* homologous genes (≥80.0% nucleotide sequence similarity) were collected from the NCBI nucleotide databases. They were mainly from the species *A. hydrophila* (58.67%, 88/150), followed by *A. veriion* (22.0%, 33/150). The rest were from *A. salmonicida* (6.0%, 9/150), other *Aeromonas* species (3.33%, 5/150), and strains of unclassified *Aeromonas* (12.70%, 19/150) (Figure 3). The nucleotide sequence similarities of the genes from *A. hydrophila* with *aac(6′)-Va* were all higher than 90.0%, while those from *A. veriion* showed lower similarities ranging between 80.0 and 90.0%. One of these homologs, a hypothetical GNAT family N-acetyltransferase (WP_158197017.1) from *A. hydrophila*, was predicted to have the highest amino acid sequence identity of 100% and similarity of 99.34% with AAC(6′)-Va, although no nucleotide sequence was available for comparison.

**Figure 4:**
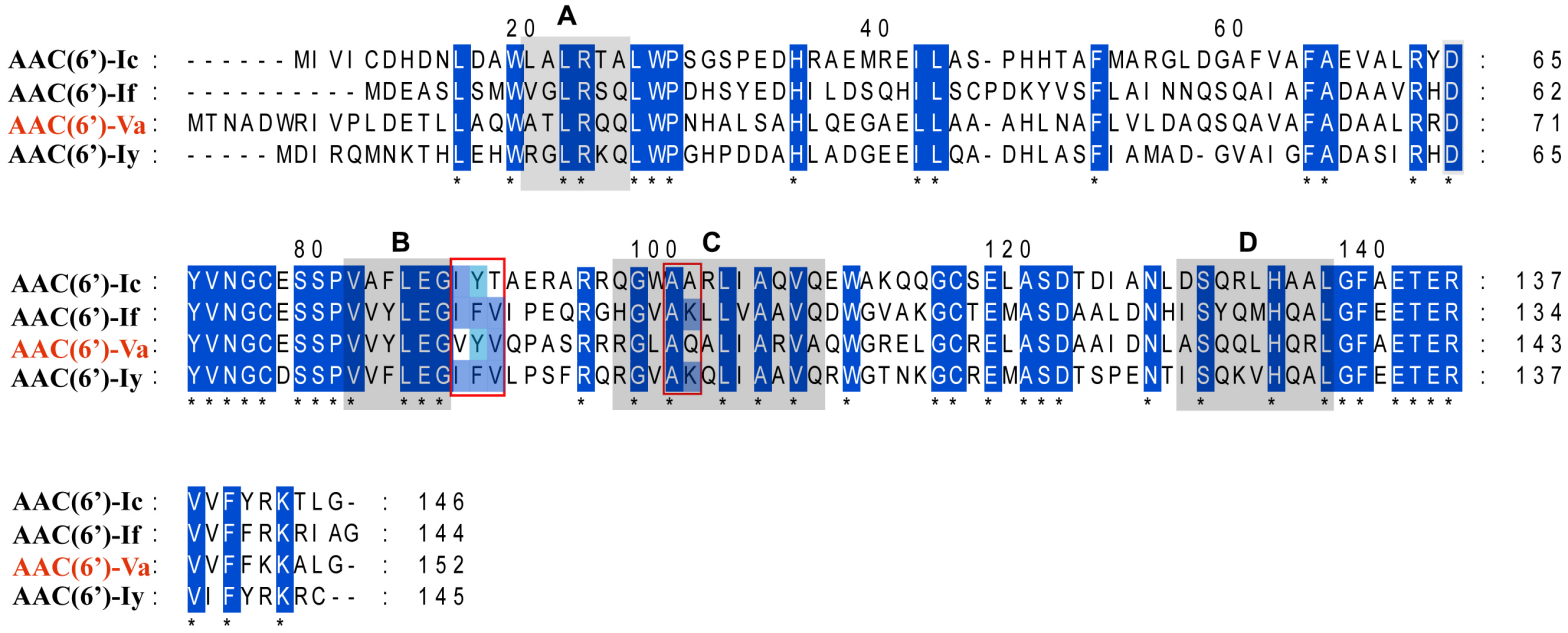
Multiple sequence alignment of the amino acid sequences of the AAC(6′)-I genes. The sequences and their accession numbers are as follows: AAC(6′)-If (CAA39038.1), AAC(6′)-Ic (AAA26549.1), and AAC(6′)-Iy (AAF03531.1). The numbers on the right correspond to the amino acid residues in each full-length protein, with fully conserved residues shown with asterisks. Motifs (**A–D**) are conserved among the entire AAC(6′) family. The red frames indicate 5 of the residues of the coenzyme A binding pocket.

**Figure 5:**
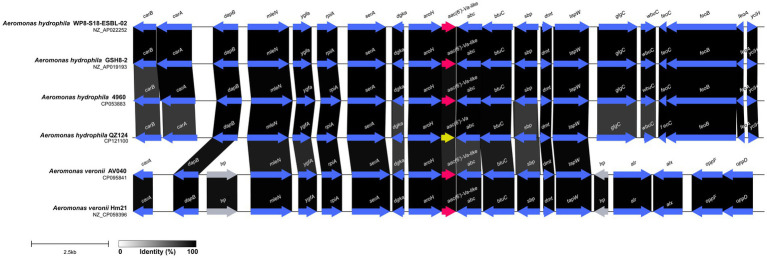
Comparative analysis of the genomic context of the *aac(6′)-Va* gene with similar sequences. Regions with ≥80.0% amino acid identity are colored gray. Accession numbers: *Aeromonas hydrophila* WP8-S18-ESBL-02 (NZ_AP022252.1), *Aeromonas hydrophila* 4,960 (CP053883.1), *Aeromonas hydrophila* GSH8-2 (NZ_AP019193.1), *Aeromonas veronii* AV040 (NZ_CP031508.1), and *Aeromonas veronii* Hm21 (NZ_CP059396.1). hp, hypothetical protein.

The multiple sequence alignment of AAC(6′) proteins demonstrated that AAC(6′)-Va shared at most 54.68, 53.58, and 53.23% identity with the three AAC(6′)-I proteins AAC(6′)-Ic (AAA26549.1), AAC(6′)-If (CAA39038.1), and AAC(6′)-Iy (AAF03531.1), respectively (Figure 4). The phylogenetic analysis of these proteins showed that AAC(6′)-Va clustered closest to AAC(6′)-Ic (Figure 2). To determine the genetic context of *aac(6′)-Va*, we intercepted 20 kb sequences with *aac(6′)-Va* and *aac(6′)-Va*-like genes (with >80.0% identity, 100% coverage to *aac(6′)-Va*) from the NCBI nonredundant nucleotide database (Figure 5). A total of 5 sequences were retrieved. No mobile genetic element was found in the adjacent regions of *aac(6′)-Va* and *aac(6′)-Va*-like genes. Three sequences from *A. hydrophila* (*A. hydrophila* WP8-S18-ESBL-02, *A. hydrophila* GSH8-2, and *A. hydrophila* 4,960) had the most similar structure (from *carB* to *yciH*) in the gene context and gene order to the sequence from this study. However, the downstream regions of the two sequences from *A. veriion* were most different from those of *A. hydrophila* QZ124.

### Kinetic parameters of AAC(6′)-Va

Investigating the acetyltransferase activity and kinetic parameters of AAC(6′)-Va revealed that the enzyme was capable of acetylating ribostamycin, kanamycin, tobramycin, sisomicin, and gentamicin, but not amikacin, out of the six aminoglycosides that were tested. Ribostamycin was observed to be the best substrate for the enzyme, with the highest catalytic efficiency [(3.35 ± 0.17) × 10^4^ M^−1^ s^−1^], whereas sisomicin was the worst [*k*_cat_/*k*_m_ ratio = (2.83 ± 0.05) × 10^3^ M^−1^ s^−1^] (Table 5). The kinetic parameters indicated that the catalytic efficiencies of the substrates for AAC(6′)-Va varied from their MIC results.

**Table 5:** Kinetic parameters of various aminoglycoside antimicrobials for AAC(6′)-Va.

Substrate	*k*_cat_ (s^−1^)^a^	*K*_m_ (μM)^a^	*k*_cat_/*K*_m_ (M^−1^ s^−1^)
Kanamycin	1.22 ± 0.31	139.95 ± 49.06	(9.03 ± 1.35) × 10^3^
Tobramycin	0.15 ± 0.02	6.04 ± 0.74	(2.44 ± 0.10) × 10^4^
Sisomicin	0.39 ± 0.02	138.43 ± 7.85	(2.83 ± 0.05) × 10^3^
Ribostamycin	0.60 ± 0.05	17.82 ± 0.51	(3.35 ± 0.17) × 10^4^
Gentamicin	0.16 ± 0.04	19.72 ± 0.87	(8.20 ± 2.41) × 10^3^
Amikacin	NA^a^	NA^a^	NA^a^

NA, no acyl transfer activity was detected.

a Values are means ± standard deviations.

## Discussion

**Figure 6:**
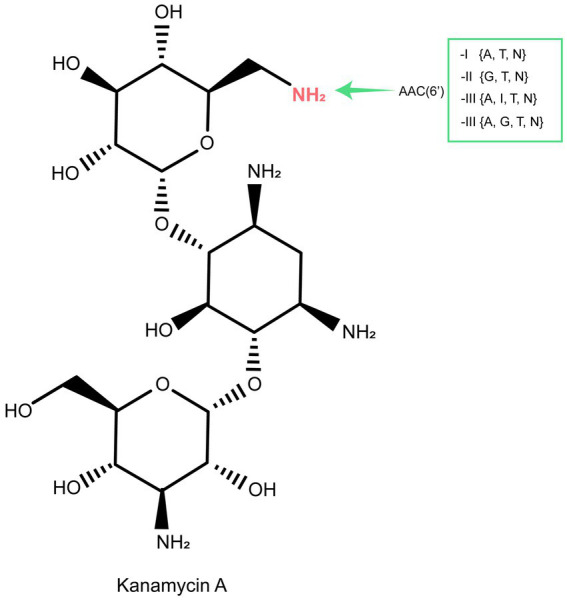
The acetylation (AAC) sites of aminoglycoside molecules by modification enzymes and the genes encoding these enzymes. Inactivation sites of the clinically useful aminoglycosides gentamicin (G), tobramycin (T), netilmicin (N), amikacin (A), or isepamicin (I) are indicated for each enzyme.

AACs are the primary mechanism by which clinical gram-negative pathogenic bacteria develop resistance to practically all clinically significant aminoglycosides (Ramirez and Tolmasky, 2010; Becker and Cooper, 2013). This resistance mechanism is complex, with over 70 AACs identified in pathogens thus far. Over 50 enzymes belonging to the AAC(6′) subclass have been identified in clinical isolates of both gram-negative and gram-positive bacteria (Ramirez and Tolmasky, 2010). AAC(6′) enzymes catalyze N-acetylation at the 6′ position of the aminoglycoside antibiotic scaffold and can be divided into four groups according to their substrate specificity: AAC(6′)-I to AAC(6′)-IV (Figure 6) (Miller et al., 1997; Över et al., 2001; Zárate et al., 2018). Genes encoding AAC(6′) were generally associated with MGEs or resistance cassettes, such as *aac(6′)-If* in *Enterobacter cloacae* (Ploy et al., 1994), *aac(6′)-Iag* in *Pseudomonas aeruginosa* (Kobayashi et al., 2013), and *aac(6′)-Ih* in *Acinetobacter baumannii* (Lambert et al., 1994), reflecting their selection in response to the use of antibiotics. However, some bacterial species also carry chromosomal genes encoding AAC(6′), such as *aac(6′)-Ic* in *Serratia marcescens* (Shaw et al., 1992), *aac(6′)-Ii* in *Enterococcus* spp. (Costa et al., 1993), and *aac(6′)-Iy* in *Salmonella enterica* and *Salmonella enteritidis* (Magnet et al., 1999).

In this work, an aminoglycoside 6′-nucleotidyltransferase gene conferring resistance to many aminoglycosides was identified to be encoded in the chromosome of a clinical *A. hydrophila* isolate. Homology analysis of AAC(6′)-Va was conducted using the NCBI nonredundant protein database and the comprehensive antibiotic resistance database (CARD, McArthur et al., 2013). The results indicated that the sequence with the highest amino acid sequence identity to AAC(6′)-Va was a 6′-nucleotidyltransferase AAC(6′)-Ic (AAA26549.1), sharing only 54.68% amino acid sequence identity. This suggests that the *aac(6′)-Va* gene is a newly identified member of the aminoglycoside 6′-nucleotidyltransferase [AAC(6′)] gene family.

Analyzing the resistance profiles of the four *aac(6′)-I* genes [including *aac(6′)-Iag*, *aac(6′)-Ic*, *aac(6′)-If*, and *aac(6′)-Iy*] with the closest evolutionary relationship to the novel aminoglycoside 6′-nucleotidyltransferase gene *aac(6′)-Va*, it was found that *aac(6′)-If*, sharing relatively higher amino acid sequence identities with *aac(6′)-Iag*, did not have any documented resistance phenotype (Kobayashi et al., 2013). The resistance profile of *aac(6′)-Va* was basically consistent with that of the other three *aac(6′)-I* genes (Shaw et al., 1992; Magnet et al., 1999; Kobayashi et al., 2013). They all conferred resistance to some aminoglycosides (e.g., gentamicin, tobramycin, and sisomicin), although the MIC levels for a few aminoglycosides were different from each other. The *aac(6′)-Va* did not show any resistance to amikacin in the antimicrobial susceptibility testing, and AAC(6′)-Va also did not show any modifying activity to amikacin in the kinetic study, which was different from the resistance phenotype of the members in the *aac(6′)-I* genes. Considering the difference in resistance profiles and the variated protein sequences between the novel gene and the *aac(6′)-I* genes, we finally designated it *aac(6′)-Va*.

The AAC(6′)-Va homologous proteins available in the NCBI nonredundant protein database were all from the genus *Aeromonas*. More than half of the proteins (58.67%, 88/150) with higher amino acid sequence similarities (with >90.0% identities, >90.0% similarity) to AAC(6′)-Va were from the same species, *A. hydrophila*, as AAC(6′)-Va in this work. From the phylogenetic analysis of AAC(6′)-Va with its homologous genes, AAC(6′)-Va was more closely related to putative AACs from *A. hydrophila*. Moreover, the *aac(6′)-Va* gene and its relatives have a conserved gene context, and they were not related to any MGEs. All of these results indicated that it might be intrinsic to this bacterial species.

## Conclusion

In this study, an aminoglycoside 6′-N-acetyltransferase, AAC(6′)-Va, that shares the highest amino acid sequence identity (54.68%) with the functionally characterized AAC(6′)-Ic and confers heightened resistance to ribostamycin is identified. Notably, the newly discovered AAC(6′)-Va exhibits different susceptibility profiles compared to other AAC(6′)-I–IV proteins. Understanding the molecular characteristics of this newly identified resistance gene will help us gain insight into the drug resistance mechanism of *Aeromonas* and related pathogenic bacteria. Given the recent increase in the prevalence of multidrug-resistant *Aeromonas*, continuous monitoring is crucial to monitor its spread.

## Data availability statement

The datasets presented in this study can be found in online repositories. The names of the repository/repositories and accession number(s) can be found in the article/Supplementary Material.

## Ethics statement

Individual patient data were not involved, and only anonymous clinical residual samples during routine hospital laboratory procedures were used in this study. This study was approved by the ethics committee of the Second Affiliated Hospital and Yuying Children’s Hospital of Wenzhou Medical University, Wenzhou, Zhejiang, China.

## Author contributions

QB, JnL, YH, and TZ: conceived and designed the experiments. GZ, LZ, YS, QC, NL, JZ, YZ, YJ, and WJ: performed the experiments. GZ, XZ, QL, JwL, XL, KL, and HZ: data analysis and interpretation. GZ, QB, JnL, and TZ: drafting of the manuscript. All authors contributed to the article and approved the submitted version.

## Footnotes

^1^http://www.softberry.com/berry.phtml?topic=bprom&group=programs&subgroup=gfindb

^2^https://web.expasy.org/protparam/

^3^https://www.ncbi.nlm.nih.gov/Structure/cdd/wrpsb.cgi

^4^https://www.python.org/
