# Supplementary material for: Identification and characterization of a novel 6′-N-aminoglycoside acetyltransferase AAC(6′)-Va from a clinical isolate of Aeromonas hydrophila
Source: Front Microbiol. 2023 Oct 18;14:1229593. doi: 10.3389/fmicb.2023.1229593 (PMC10619662; doi:10.3389/fmicb.2023.1229593)
Supplement: Supplementary file 1 [file Table_1.DOCX]

Table S1. The *aac(6’)-Va* gene homology analysis results of 150 *Aeromonas* isolates.

| Gene | Amino acids | Ref sequences | Identity (%) | similarity (%) | Accession No. |
| --- | --- | --- | --- | --- | --- |
| *aac(6’)-Va* -*like* | 151 | GNAT family N-acetyltransferase [*Aeromonas hydrophila*] | 99.34 | 99.34 | WP_253626170.1 |
| *aac(6’)-Va* -like | 151 | GNAT family N-acetyltransferase [*Aeromonas hydrophila*] | 100 | 99.34 | WP_158197017.1 |
| *aac(6’)-Va* -like | 151 | GNAT family N-acetyltransferase [*Aeromonas hydrophila*] | 99.34 | 98.68 | WP_077096780.1 |
| *aac(6’)-Va* -like | 151 | GNAT family N-acetyltransferase [*Aeromonas hydrophila*] | 100 | 98.68 | WP_029300039.1 |
| *aac(6’)-Va* -like | 149 | GNAT family N-acetyltransferase [*Aeromonas hydrophila*] | 99.34 | 98.68 | BCO13219.1 |
| *aac(6’)-Va* -like | 151 | GNAT family N-acetyltransferase [*Aeromonas hydrophila*] | 99.34 | 98.68 | WP_201355748.1 |
| *aac(6’)-Va* -like | 151 | GNAT family N-acetyltransferase [*Aeromonas hydrophila*] | 100 | 98.03 | WP_257689562.1 |
| *aac(6’)-Va* -like | 151 | GNAT family N-acetyltransferase [*Aeromonas hydrophila*] | 99.34 | 98.03 | WP_203091876.1 |
| *aac(6’)-Va* -like | 151 | GNAT family N-acetyltransferase [*Aeromonas hydrophila*] | 98.68 | 98.03 | WP_139388066.1 |
| *aac(6’)-Va* -like | 151 | GNAT family N-acetyltransferase [*Aeromonas*] | 99.34 | 97.37 | WP_202004133.1 |
| *aac(6’)-Va* -like | 151 | GNAT family N-acetyltransferase [*Aeromonas hydrophila*] | 98.68 | 97.37 | WP_130632068.1 |
| *aac(6’)-Va* -like | 149 | GNAT family N-acetyltransferase [*Aeromonas hydrophila*] | 98.68 | 97.37 | WP_257620874.1 |
| *aac(6’)-Va* -like | 151 | GNAT family N-acetyltransferase [*Aeromonas hydrophila*] | 98.68 | 97.37 | MBW3834639.1 |
| *aac(6’)-Va* -like | 149 | GNAT family N-acetyltransferase [*Aeromonas hydrophila*] | 98.68 | 96.71 | GKQ99651.1 |
| *aac(6’)-Va* -like | 151 | GNAT family N-acetyltransferase [*Aeromonas hydrophila*] | 98.68 | 97.37 | WP_029303895.1 |
| *aac(6’)-Va* -like | 151 | GNAT family N-acetyltransferase [*Aeromonas hydrophila*] | 98.68 | 98.03 | EGX6953910.1 |
| *aac(6’)-Va* -like | 151 | GNAT family N-acetyltransferase [*Aeromonas hydrophila*] | 98.68 | 96.71 | WP_244090241.1 |
| *aac(6’)-Va* -like | 151 | GNAT family N-acetyltransferase [*Aeromonas*] | 98.68 | 96.71 | WP_139389996.1 |
| *aac(6’)-Va* -like | 151 | GNAT family N-acetyltransferase [*Aeromonas hydrophila*] | 99.34 | 98.03 | WP_215783761.1 |
| *aac(6’)-Va* -like | 151 | GNAT family N-acetyltransferase [*Aeromonas hydrophila*] | 99.34 | 97.37 | WP_219253414.1 |
| *aac(6’)-Va* -like | 151 | GNAT family N-acetyltransferase [*Aeromonas hydrophila*] | 98.68 | 96.71 | WP_011706546.1 |
| *aac(6’)-Va* -like | 151 | GNAT family N-acetyltransferase [*Aeromonas hydrophila*] | 98.68 | 96.71 | WP_268030485.1 |
| *aac(6’)-Va* -like | 151 | GNAT family N-acetyltransferase [*Aeromonas sp*. QDB54] | 98.03 | 97.37 | WP_270802529.1 |
| *aac(6’)-Va* -like | 151 | GNAT family N-acetyltransferase [*Aeromonas hydrophila*] | 98.68 | 96.71 | WP_226031523.1 |
| *aac(6’)-Va* -like | 151 | GNAT family N-acetyltransferase [*Aeromonas hydrophila*] | 98.68 | 96.05 | WP_044800321.1 |
| *aac(6’)-Va* -like | 151 | GNAT family N-acetyltransferase [*Aeromonas hydrophila*] | 98.03 | 96.05 | WP_226031950.1 |
| *aac(6’)-Va* -like | 151 | GNAT family N-acetyltransferase [*Aeromonas hydrophila*] | 98.68 | 96.71 | WP_201974788.1 |
| *aac(6’)-Va* -like | 151 | GNAT family N-acetyltransferase [*Aeromonas hydrophila*] | 98.03 | 97.37 | WP_124251999.1 |
| *aac(6’)-Va* -like | 151 | MULTISPECIES: GNAT family N-acetyltransferase [*Aeromonas*] | 98.68 | 96.05 | WP_103828717.1 |
| *aac(6’)-Va* -like | 151 | GNAT family N-acetyltransferase [*Aeromonas hydrophila*] | 98.03 | 96.71 | WP_203133953.1 |
| *aac(6’)-Va* -like | 151 | GNAT family N-acetyltransferase [*Aeromonas hydrophila*] | 98.68 | 97.37 | WP_257685387.1 |
| *aac(6’)-Va* -like | 151 | GNAT family N-acetyltransferase [*Aeromonas hydrophila*] | 98.68 | 96.05 | WP_252476710.1 |
| *aac(6’)-Va* -like | 151 | GNAT family N-acetyltransferase [*Aeromonas hydrophila*] | 98.68 | 97.37 | HAT6346618.1 |
| *aac(6’)-Va* -like | 151 | GNAT family N-acetyltransferase [*Aeromonas hydrophila*] | 98.03 | 96.05 | WP_076361398.1 |
| *aac(6’)-Va* -like | 151 | GNAT family N-acetyltransferase [*Aeromonas hydrophila*] | 98.03 | 96.71 | QWL74342.1 |
| *aac(6’)-Va* -like | 151 | GNAT family N-acetyltransferase [*Aeromonas hydrophila*] | 98.03 | 96.05 | WP_241872128.1 |
| *aac(6’)-Va* -like | 151 | GNAT family N-acetyltransferase [*Aeromonas hydrophila*] | 98.03 | 96.71 | WP_224480762.1 |
| *aac(6’)-Va* -like | 151 | GNAT family N-acetyltransferase [*Aeromonas hydrophila*] | 98.68 | 96.05 | WP_042067834.1 |
| *aac(6’)-Va* -like | 151 | GNAT family N-acetyltransferase [*Aeromonas hydrophila*]] | 98.03 | 96.71 | WP_101616776.1 |
| *aac(6’)-Va* -like | 151 | GNAT family N-acetyltransferase [*Aeromonas hydrophila*] | 98.03 | 96.71 | WP_242798416.1 |
| *aac(6’)-Va* -like | 151 | GNAT family N-acetyltransferase [*Aeromonas hydrophila*] | 98.68 | 96.05 | WP_118880903.1 |
| *aac(6’)-Va* -like | 151 | MULTISPECIES: GNAT family N-acetyltransferase [*Aeromonas*] | 96.71 | 95.40 | WP_073350794.1 |
| *aac(6’)-Va* -like | 151 | GNAT family N-acetyltransferase [*Aeromonas hydrophila*] | 98.03 | 95.40 | HAU4890392.1 |
| *aac(6’)-Va* -like | 151 | GNAT family N-acetyltransferase [*Aeromonas hydrophila*] | 98.68 | 96.05 | WP_106552860.1 |
| *aac(6’)-Va* -like | 151 | GNAT family N-acetyltransferase [*Aeromonas hydrophila*] | 97.37 | 96.71 | WP_045527313.1 |
| *aac(6’)-Va* -like | 151 | GNAT family N-acetyltransferase [*Aeromonas hydrophila*] | 98.03 | 95.40 | WP_045789461.1 |
| *aac(6’)-Va* -like | 149 | GNAT family N-acetyltransferase [*Aeromonas hydrophila*] | 98.03 | 96.71 | CAD7546396.1 |
| *aac(6’)-Va* -like | 151 | GNAT family N-acetyltransferase [*Aeromonas sp.* BC14] | 98.03 | 96.05 | WP_268015252.1 |
| *aac(6’)-Va* -like | 151 | GNAT family N-acetyltransferase [*Aeromonas hydrophila*] | 98.68 | 95.40 | WP_043165558.1 |
| *aac(6’)-Va* -like | 151 | GNAT family N-acetyltransferase [*Aeromonas hydrophila*] | 98.03 | 96.71 | WP_043123980.1 |
| *aac(6’)-Va* -like | 151 | GNAT family N-acetyltransferase [*Aeromonas hydrophila*] | 98.03 | 94.74 | WP_017410873.1 |
| *aac(6’)-Va* -like | 151 | GNAT family N-acetyltransferase [*Aeromonas hydrophila*] | 97.37 | 94.74 | WP_102988445.1 |
| *aac(6’)-Va* -like | 151 | GNAT family N-acetyltransferase [*Aeromonas hydrophila*] | 96.71 | 95.40 | WP_081304554.1 |
| *aac(6’)-Va* -like | 151 | GNAT family N-acetyltransferase [*Aeromonas hydrophila*] | 98.03 | 96.05 | HAU4907387.1 |
| *aac(6’)-Va* -like | 151 | GNAT family N-acetyltransferase [*Aeromonas hydrophila*] | 98.68 | 95.40 | WP_254201535.1 |
| *aac(6’)-Va* -like | 151 | MULTISPECIES: GNAT family N-acetyltransferase [unclassified *Aeromonas*] | 96.71 | 96.05 | WP_259236739.1 |
| *aac(6’)-Va* -like | 151 | GNAT family N-acetyltransferase [*Aeromonas sp.* A35_P] | 96.71 | 94.74 | WP_094697964.1 |
| *aac(6’)-Va* -like | 151 | GNAT family N-acetyltransferase [*Aeromonas hydrophila*] | 98.03 | 96.05 | WP_024946384.1 |
| *aac(6’)-Va* -like | 151 | GNAT family N-acetyltransferase [*Aeromonas hydrophila*] | 98.03 | 96.05 | WP_041217812.1 |
| *aac(6’)-Va* -like | 151 | GNAT family N-acetyltransferase [*Aeromonas sp.* 2692-1] | 98.03 | 95.40 | WP_113994361.1 |
| *aac(6’)-Va* -like | 151 | GNAT family N-acetyltransferase [*Aeromonas hydrophila*] | 97.37 | 94.74 | CAD7543310.1 |
| *aac(6’)-Va* -like | 151 | GNAT family N-acetyltransferase [*Aeromonas sp.* 2692-1] | 97.37 | 95.40 | WP_171280770.1 |
| *aac(6’)-Va* -like | 151 | GNAT family N-acetyltransferase [*Aeromonas hydrophila*] | 98.03 | 95.40 | WP_206818106.1 |
| *aac(6’)-Va* -like | 151 | GNAT family N-acetyltransferase [*Aeromonas hydrophila*] | 97.37 | 95.40 | GJC06758.1 |
| *aac(6’)-Va* -like | 151 | GNAT family N-acetyltransferase [*Aeromonas hydrophila*] | 97.37 | 94.74 | WP_252452782.1 |
| *aac(6’)-Va* -like | 151 | GNAT family N-acetyltransferase [*Aeromonas hydrophila*] | 97.37 | 94.74 | WP_016351195.1 |
| *aac(6’)-Va* -like | 151 | MULTISPECIES: GNAT family N-acetyltransferase [*Aeromonas*] | 97.37 | 95.40 | WP_223951743.1 |
| *aac(6’)-Va* -like | 151 | 1-152 GNAT family N-acetyltransferase [*Aeromonas hydrophila*] | 97.37 | 95.40 | WP_039216005.1 |
| *aac(6’)-Va* -like | 151 | 1-152 GNAT family N-acetyltransferase [*Aeromonas hydrophila*] | 97.37 | 95.40 | WP_259232386.1 |
| *aac(6’)-Va* -like | 149 | 3-154 aminoglycoside N(6')-acetyltransferase [*Aeromonas hydrophila*] | 98.03 | 95.40 | AZU48606.1 |
| *aac(6’)-Va* -like | 151 | 1-152 GNAT family N-acetyltransferase [*Aeromonas hydrophila*] | 98.03 | 95.40 | WP_166463948.1 |
| *aac(6’)-Va* -like | 151 | 1-152 GNAT family N-acetyltransferase [*Aeromonas hydrophila*] | 97.37 | 95.40 | WP_201905089.1 |
| *aac(6’)-Va* -like | 151 | 1-152 GNAT family N-acetyltransferase [*Aeromonas hydrophila*] | 98.03 | 95.40 | WP_135319462.1 |
| *aac(6’)-Va* -like | 151 | 1-152 GNAT family N-acetyltransferase [*Aeromonas hydrophila*] | 97.37 | 96.05 | WP_060390213.1 |
| *aac(6’)-Va* -like | 151 | 1-151 GNAT family N-acetyltransferase [*Aeromonas hydrophila*] | 98.01 | 96.03 | EIS3738154.1 |
| *aac(6’)-Va* -like | 149 | 3-154 aminoglycoside N(6')-acetyltransferase type 1 [*Aeromonas hydrophila*] | 98.03 | 95.40 | BDC81880.1 |
| *aac(6’)-Va* -like | 151 | 1-152 MULTISPECIES: GNAT family N-acetyltransferase [*Aeromonas*] | 97.37 | 95.40 | WP_182859849.1 |
| *aac(6’)-Va* -like | 151 | 1-152 GNAT family N-acetyltransferase [*Aeromonas hydrophila*] | 97.37 | 95.40 | WP_139748766.1 |
| *aac(6’)-Va* -like | 151 | 1-152 GNAT family N-acetyltransferase [*Aeromonas hydrophila*] | 98.03 | 95.40 | WP_236766719.1 |
| *aac(6’)-Va* -like | 149 | 3-154 aminoglycoside N(6')-acetyltransferase type 1 [*Aeromonas hydrophila*] | 97.37 | 95.40 | BCK62714.1 |
| *aac(6’)-Va* -like | 151 | 1-152 GNAT family N-acetyltransferase [*Aeromonas rivuli*] | 96.05 | 95.40 | WP_042043669.1 |
| *aac(6’)-Va* -like | 151 | 1-152 GNAT family N-acetyltransferase [*Aeromonas hydrophila*] | 96.71 | 94.08 | EIS3743776.1 |
| *aac(6’)-Va* -like | 151 | 1-151 GNAT family N-acetyltransferase [*Aeromonas hydrophila*] | 98.01 | 95.36 | MBX9565754.1 |
| *aac(6’)-Va* -like | 151 | 1-152 GNAT family N-acetyltransferase [*Aeromonas dhakensis*] | 98.03 | 93.42 | WP_208201289.1 |
| *aac(6’)-Va* -like | 151 | 1-152 GNAT family N-acetyltransferase [*Aeromonas hydrophila*] | 96.05 | 93.42 | WP_139392836.1 |
| *aac(6’)-Va* -like | 151 | 1-152 GNAT family N-acetyltransferase [*Aeromonas hydrophila*] | 96.71 | 93.42 | WP_187268410.1 |
| *aac(6’)-Va* -like | 151 | 1-157 GNAT family N-acetyltransferase [*Aeromonas hydrophila*] | 94.27 | 91.72 | WP_139704141.1 |
| *aac(6’)-Va* -like | 151 | 1-152 GNAT family N-acetyltransferase [*Aeromonas hydrophila*] | 96.05 | 93.42 | WP_194758751.1 |
| *aac(6’)-Va* -like | 151 | 1-152 GNAT family N-acetyltransferase [*Aeromonas hydrophila*] | 95.39 | 91.45 | WP_101148932.1 |
| *aac(6’)-Va* -like | 151 | 1-152 aminoglycoside 6'-acetyltransferase [*Aeromonas sp.* CU5] | 94.08 | 89.47 | ATL95130.1 |
| *aac(6’)-Va* -like | 149 | 3-154 GNAT family N-acetyltransferase [*Aeromonas sp.* CU5] | 94.08 | 89.47 | WP_253860407.1 |
| *aac(6’)-Va* -like | 149 | 3-154 MULTISPECIES: GNAT family N-acetyltransferase [*Aeromonas*] | 94.08 | 89.47 | WP_256028903.1 |
| *aac(6’)-Va* -like | 151 | 1-152 MULTISPECIES: GNAT family N-acetyltransferase [*Aeromonas*] | 94.08 | 88.82 | WP_047437493.1 |
| *aac(6’)-Va* -like | 151 | 1-145 aminoglycoside 6'-acetyltransferase [*Aeromonas hydrophila*] | 97.93 | 95.86 | OCA61481.1 |
| *aac(6’)-Va* -like | 151 | 1-152 GNAT family N-acetyltransferase [*Aeromonas veronii*] | 94.08 | 88.82 | WP_088869320.1 |
| *aac(6’)-Va* -like | 151 | 1-152 GNAT family N-acetyltransferase [*Aeromonas veronii*] | 94.08 | 88.82 | WP_219295498.1 |
| *aac(6’)-Va* -like | 151 | 1-152 GNAT family N-acetyltransferase [*Aeromonas veronii*] | 94.08 | 88.16 | WP_181221767.1 |
| *aac(6’)-Va* -like | 151 | 1-152 GNAT family N-acetyltransferase [*Aeromonas veronii*] | 93.42 | 88.16 | WP_181223677.1 |
| *aac(6’)-Va* -like | 151 | 1-152 GNAT family N-acetyltransferase [*Aeromonas veronii*] | 94.08 | 88.16 | WP_139477728.1 |
| *aac(6’)-Va* -like | 151 | 1-152 MULTISPECIES: GNAT family N-acetyltransferase [*Aeromonas*] | 94.08 | 88.16 | WP_139428413.1 |
| *aac(6’)-Va* -like | 151 | 1-152 GNAT family N-acetyltransferase [*Aeromonas veronii*] | 93.42 | 88.16 | WP_139473593.1 |
| *aac(6’)-Va* -like | 151 | 1-147 GNAT family N-acetyltransferase [*Aeromonas hydrophila*] | 94.08 | 92.11 | WP_049045557.1 |
| *aac(6’)-Va* -like | 151 | 1-152 GNAT family N-acetyltransferase [*Aeromonas veronii*] | 94.08 | 87.50 | WP_107682329.1 |
| *aac(6’)-Va* -like | 151 | 1-152 GNAT family N-acetyltransferase [*Aeromonas veronii*] | 93.42 | 88.16 | WP_201950316.1 |
| *aac(6’)-Va* -like | 151 | 1-152 MULTISPECIES: GNAT family N-acetyltransferase [*Aeromonas*] | 93.42 | 88.16 | WP_139424995.1 |
| *aac(6’)-Va* -like | 151 | 1-152 MULTISPECIES: GNAT family N-acetyltransferase [unclassified *Aeromonas*] | 93.42 | 88.16 | WP_216961920.1 |
| *aac(6’)-Va* -like | 151 | 1-152 GNAT family N-acetyltransferase [*Aeromonas veronii*] | 93.42 | 88.16 | WP_005337645.1 |
| *aac(6’)-Va* -like | 151 | 1-152 GNAT family N-acetyltransferase [*Aeromonas veronii*] | 93.42 | 87.50 | WP_236325080.1 |
| *aac(6’)-Va* -like | 151 | 1-152 GNAT family N-acetyltransferase [*Aeromonas veronii*] | 93.42 | 87.50 | WP_005361592.1 |
| *aac(6’)-Va* -like | 149 | 3-154 aminoglycoside 6'-N-acetyltransferase I [*Aeromonas veronii*] | 93.42 | 88.16 | SIR50314.1 |
| *aac(6’)-Va* -like | 151 | 1-152 GNAT family N-acetyltransferase [*Aeromonas veronii*] | 93.42 | 88.16 | WP_076495566.1 |
| *aac(6’)-Va* -like | 151 | 1-152 GNAT family N-acetyltransferase [*Aeromonas veronii*] | 93.42 | 87.50 | WP_139464053.1 |
| *aac(6’)-Va* -like | 151 | 1-152 GNAT family N-acetyltransferase [*Aeromonas veronii*] | 93.42 | 87.50 | WP_194495753.1 |
| *aac(6’)-Va* -like | 151 | 1-152 GNAT family N-acetyltransferase [*Aeromonas veronii*] | 93.42 | 87.50 | WP_267517549.1 |
| *aac(6’)-Va* -like | 151 | 1-152 GNAT family N-acetyltransferase [*Aeromonas veronii*] | 93.42 | 86.84 | WP_245148262.1 |
| *aac(6’)-Va* -like | 151 | 1-152 GNAT family N-acetyltransferase [*Aeromonas veronii*] | 93.42 | 86.84 | WP_139442557.1 |
| *aac(6’)-Va* -like | 151 | 1-152 GNAT family N-acetyltransferase [*Aeromonas veronii*] | 93.42 | 86.84 | WP_263085955.1 |
| *aac(6’)-Va* -like | 151 | 1-152 MULTISPECIES: GNAT family N-acetyltransferase [*Aeromonas*] | 93.42 | 86.84 | WP_199427220.1 |
| *aac(6’)-Va* -like | 151 | 1-152 GNAT family N-acetyltransferase [*Aeromonas veronii*] | 92.11 | 86.18 | WP_101530488.1 |
| *aac(6’)-Va* -like | 151 | 1-152 GNAT family N-acetyltransferase [*Aeromonas veronii*] | 92.76 | 86.84 | WP_257701297.1 |
| *aac(6’)-Va* -like | 151 | 1-152 GNAT family N-acetyltransferase [*Aeromonas veronii*] | 93.42 | 86.84 | WP_245098292.1 |
| *aac(6’)-Va* -like | 151 | 1-152 GNAT family N-acetyltransferase [*Aeromonas veronii*] | 92.76 | 86.84 | WP_201973996.1 |
| *aac(6’)-Va* -like | 151 | 1-152 GNAT family N-acetyltransferase [*Aeromonas veronii*] | 92.76 | 86.84 | WP_216977954.1 |
| *aac(6’)-Va* -like | 151 | 1-152 GNAT family N-acetyltransferase [*Aeromonas veronii*] | 92.76 | 86.84 | WP_167567558.1 |
| *aac(6’)-Va* -like | 150 | 1-151 MULTISPECIES: GNAT family N-acetyltransferase [unclassified *Aeromonas*] | 93.38 | 87.42 | WP_108540769.1 |
| *aac(6’)-Va* -like | 151 | 1-152 GNAT family N-acetyltransferase [*Aeromonas veronii]* | 92.76 | 86.18 | WP_021230627.1 |
| *aac(6’)-Va* -like | 151 | 1-152 GNAT family N-acetyltransferase [*Aeromonas veronii*] | 92.11 | 86.18 | WP_005353112.1 |
| *aac(6’)-Va* -like | 151 | 1-138 GNAT family N-acetyltransferase [*Aeromonas hydrophila*] | 98.55 | 96.38 | HAT1553723.1 |
| *aac(6’)-Va* -like | 151 | 1-152 GNAT family N-acetyltransferase [*Aeromonas veronii*] | 91.45 | 85.53 | WP_213388878.1 |
| *aac(6’)-Va* -like | 151 | 1-152 GNAT family N-acetyltransferase [*Aeromonas salmonicida*] | 90.79 | 85.53 | WP_058394148.1 |
| *aac(6’)-Va* -like | 151 | 1-152 GNAT family N-acetyltransferase [*Aeromonas veronii*] | 90.13 | 83.55 | WP_236321927.1 |
| *aac(6’)-Va* -like | 151 | 1-152 GNAT family N-acetyltransferase [*Aeromonas salmonicida*] | 89.47 | 84.87 | WP_265462240.1 |
| *aac(6’)-Va* -like | 151 | 1-152 GNAT family N-acetyltransferase [*Aeromonas salmonicida*] | 90.13 | 84.21 | WP_265467151.1 |
| *aac(6’)-Va* -like | 151 | 1-152 GNAT family N-acetyltransferase [*Aeromonas veronii*] | 89.47 | 83.55 | WP_113739211.1 |
| *aac(6’)-Va* -like | 151 | 1-152 GNAT family N-acetyltransferase [*Aeromonas salmonicida*] | 90.13 | 83.55 | WP_059169335.1 |
| *aac(6’)-Va* -like | 151 | 1-152 aminoglycoside 6'-acetyltransferase [*Aeromonas salmonicida*] | 89.47 | 82.90 | HBL03849.1 |
| *aac(6’)-Va* -like | 151 | 1-152 GNAT family N-acetyltransferase [*Aeromonas salmonicida*] | 89.47 | 83.55 | WP_265432030.1 |
| *aac(6’)-Va* -like | 151 | 1-152 GNAT family N-acetyltransferase [*Aeromonas piscicola*] | 88.82 | 83.55 | WP_264303548.1 |
| *aac(6’)-Va* -like | 151 | 1-152 GNAT family N-acetyltransferase [*Aeromonas salmonicida*] | 89.47 | 83.55 | WP_125601800.1 |
| *aac(6’)-Va* -like | 151 | 1-152 GNAT family N-acetyltransferase [*Aeromonas piscicola*] | 88.16 | 82.90 | WP_042866208.1 |
| *aac(6’)-Va* -like | 151 | 1-145 aminoglycoside 6'-acetyltransferase [*Aeromonas veronii*] | 93.10 | 86.90 | OKP40008.1 |
| *aac(6’)-Va* -like | 151 | 1-152 GNAT family N-acetyltransferase [*Aeromonas salmonicida*] | 89.47 | 83.55 | WP_087755426.1 |
| *aac(6’)-Va* -like | 151 | 1-147 GNAT family N-acetyltransferase [*Aeromonas veronii*] | 90.13 | 84.87 | WP_201989145.1 |
| *aac(6’)-Va* -like | 151 | 1-152 aminoglycoside 6'-acetyltransferase [*Aeromonas piscicola*] | 88.16 | 82.24 | OCA65120.1 |
| *aac(6’)-Va* -like | 145 | 1-146 GNAT family N-acetyltransferase, partial [*Aeromonas sp.* ANP5] | 91.78 | 85.62 | WP_069526715.1 |
| *aac(6’)-Va* -like | 144 | 1-145 GNAT family N-acetyltransferase, partial [*Aeromonas sp.* ANNP30] | 92.41 | 86.21 | WP_069566706.1 |
| *aac(6’)-Va* -like | 151 | 1-152 GNAT family N-acetyltransferase [*Aeromonas salmonicida*] | 88.82 | 82.24 | WP_155603935.1 |
| *aac(6’)-Va* -like | 151 | 1-146 GNAT family N-acetyltransferase [*Aeromonas veronii*] | 89.47 | 83.55 | WP_157160482.1 |
| *aac(6’)-Va* -like | 151 | 1-152 GNAT family N-acetyltransferase [*Aeromonas tecta*] | 87.50 | 81.58 | WP_050716733.1 |
| *aac(6’)-Va* -like | 151 | 1-152 GNAT family N-acetyltransferase [*Aeromonas allosaccharophila*] | 86.84 | 80.26 | WP_139708959.1 |
