# Supplementary figures and images for: Identification and characterization of a novel 6′-N-aminoglycoside acetyltransferase AAC(6′)-Va from a clinical isolate of Aeromonas hydrophila
Source: Front Microbiol. 2023 Oct 18;14:1229593. doi: 10.3389/fmicb.2023.1229593 (PMC10619662; doi:10.3389/fmicb.2023.1229593)

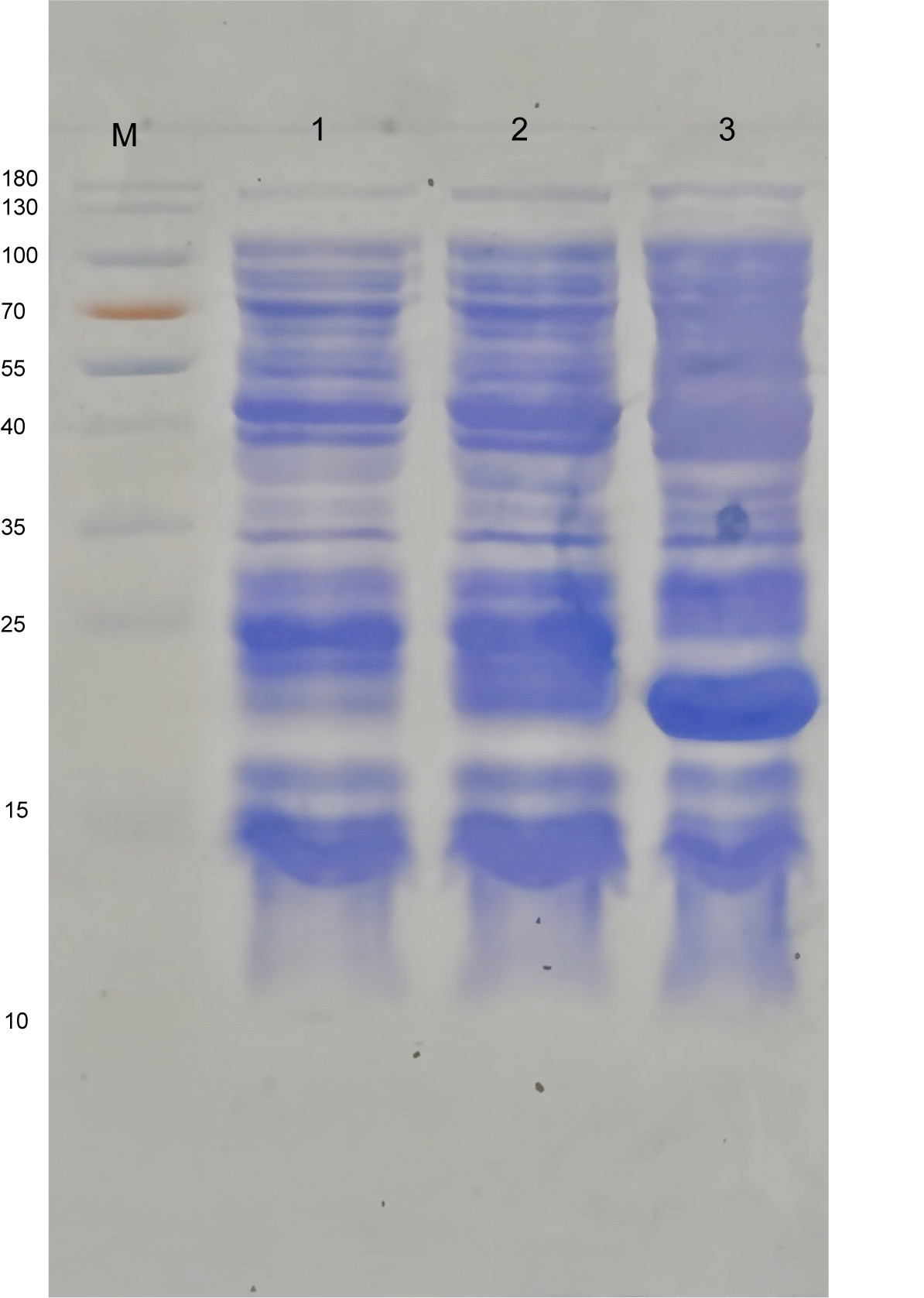

Supplement: Supplementary file 2 [file Image_1.JPEG]

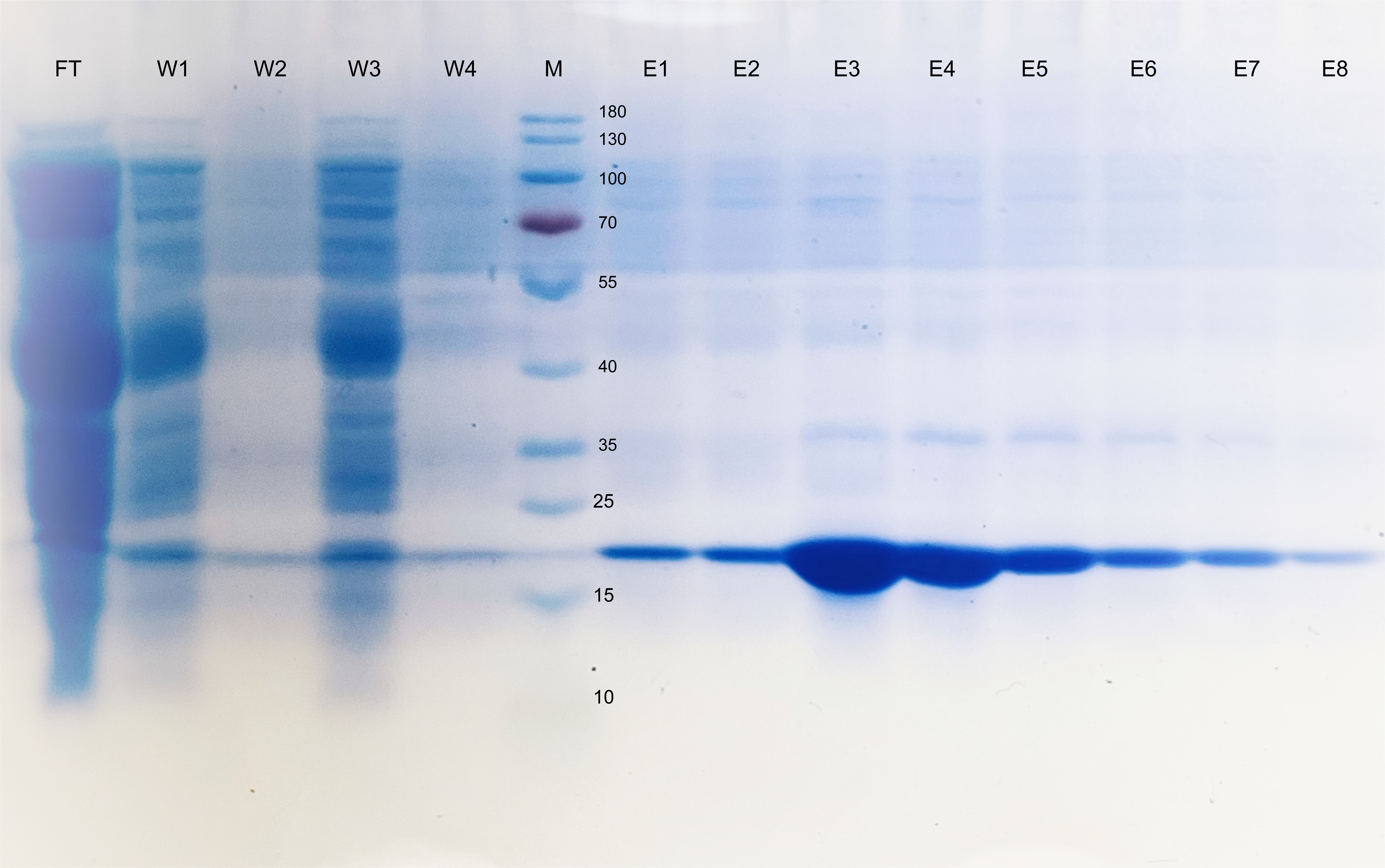

Supplement: Supplementary file 3 [file Image_2.JPEG]

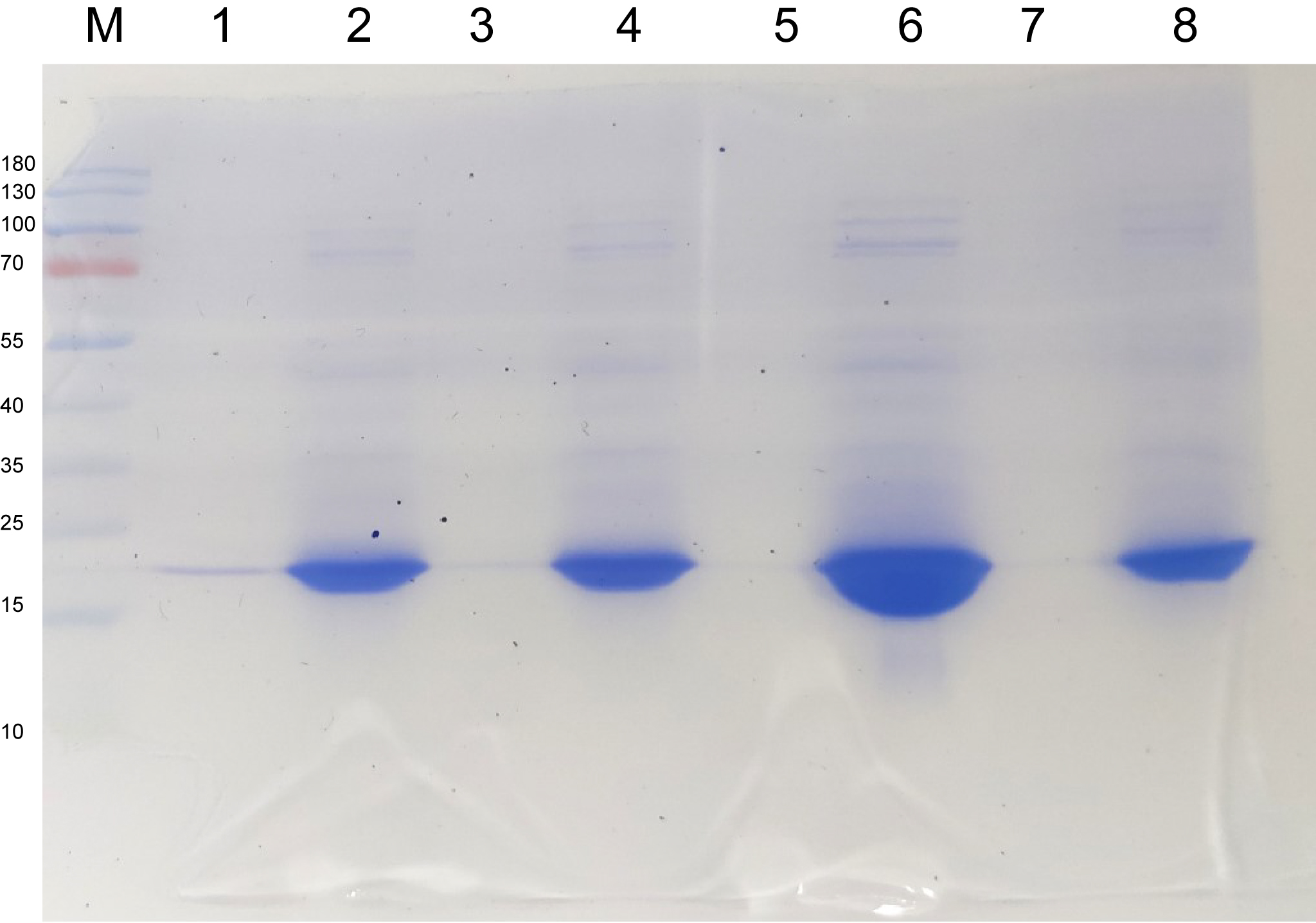

Supplement: Supplementary file 4 [file Image_3.JPEG]

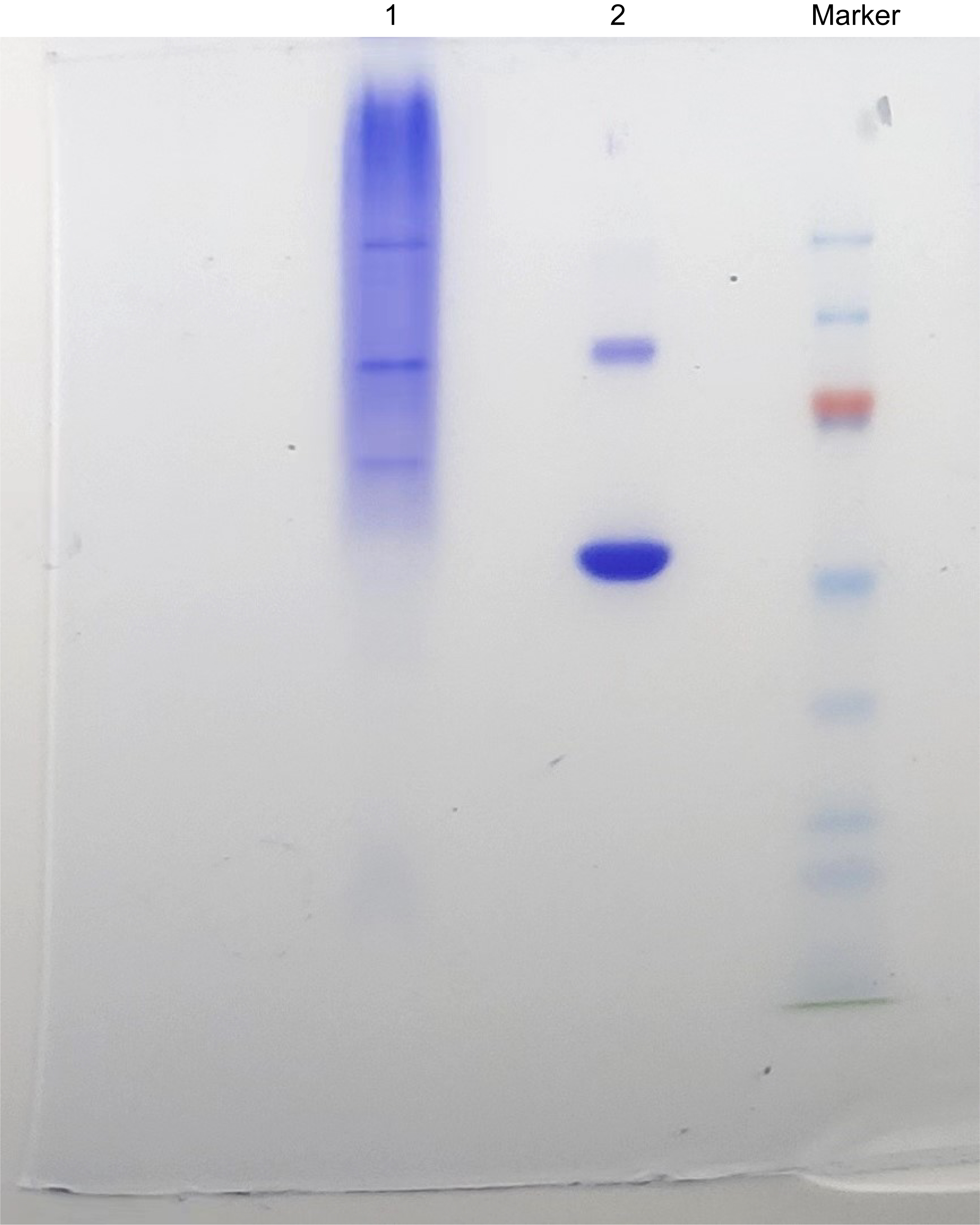

Supplement: Supplementary file 5 [file Image_4.JPEG]
